# Predicting adult neuroscience intensive care unit admission from emergency department triage using a retrospective, tabular-free text machine learning approach

**DOI:** 10.1038/s41598-021-80985-3

**Published:** 2021-01-14

**Authors:** Eyal Klang, Benjamin R. Kummer, Neha S. Dangayach, Amy Zhong, M. Arash Kia, Prem Timsina, Ian Cossentino, Anthony B. Costa, Matthew A. Levin, Eric K. Oermann

**Affiliations:** 1 Institute for Healthcare Delivery Science, Icahn School of Medicine at Mount Sinai, New York, NY USA; 2 Department of Neurology, Icahn School of Medicine at Mount Sinai, One Gustave Levy Place, Box 1137, New York, NY USA; 3 Clinical Informatics, Mount Sinai Health System, New York, NY USA; 4 Department of Neurosurgery, Icahn School of Medicine at Mount Sinai, New York, NY USA; 5 Icahn School of Medicine at Mount Sinai, New York, NY USA; 6 Department of Anesthesiology, Perioperative and Pain Medicine, Icahn School of Medicine at Mount Sinai, New York, NY USA

**Keywords:** Neurology, Predictive medicine

## Abstract

Early admission to the neurosciences intensive care unit (NSICU) is associated with improved patient outcomes. Natural language processing offers new possibilities for mining free text in electronic health record data. We sought to develop a machine learning model using both tabular and free text data to identify patients requiring NSICU admission shortly after arrival to the emergency department (ED). We conducted a single-center, retrospective cohort study of adult patients at the Mount Sinai Hospital, an academic medical center in New York City. All patients presenting to our institutional ED between January 2014 and December 2018 were included. Structured (tabular) demographic, clinical, bed movement record data, and free text data from triage notes were extracted from our institutional data warehouse. A machine learning model was trained to predict likelihood of NSICU admission at 30 min from arrival to the ED. We identified 412,858 patients presenting to the ED over the study period, of whom 1900 (0.5%) were admitted to the NSICU. The daily median number of ED presentations was 231 (IQR 200–256) and the median time from ED presentation to the decision for NSICU admission was 169 min (IQR 80–324). A model trained only with text data had an area under the receiver-operating curve (AUC) of 0.90 (95% confidence interval (CI) 0.87–0.91). A structured data-only model had an AUC of 0.92 (95% CI 0.91–0.94). A combined model trained on structured and text data had an AUC of 0.93 (95% CI 0.92–0.95). At a false positive rate of 1:100 (99% specificity), the combined model was 58% sensitive for identifying NSICU admission. A machine learning model using structured and free text data can predict NSICU admission soon after ED arrival. This may potentially improve ED and NSICU resource allocation. Further studies should validate our findings.

## Introduction

**Figure 1:**
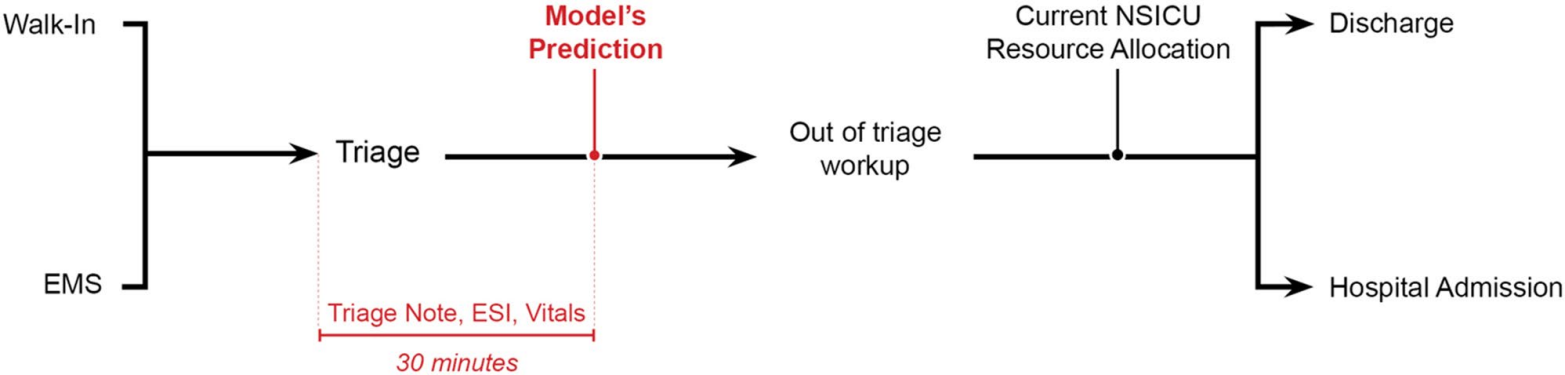
Diagram of patient flow through our institutional emergency department. Patient movement occurs from far left (arrival) to far right (final disposition). Current-state triage point to the neuroscience intensive care unit appears to the right. Proposed triage point and predictive time window appear in red. *EMS* emergency medical services, *ESI* emergency severity index, *NSICU* neuroscience intensive care unit.

Critically ill neurological patients benefit from care in the neurosciences intensive care unit (NSICU)^1–4^. Delays in reaching the NSICU are associated with adverse clinical outcomes^5^. Moreover, ED overcrowding is a well-recognized hindrance to patient care that is associated with increased length of stay and hospitalization costs^6–10^. NSICU care is both time sensitive and costly^11, 12^, and use of the NSICU requires prompt and judicious decision-making. However, identification of patients requiring NSICU care may occur downstream from initial triage (as in Fig. 1), potentially contributing to harmful and costly care delays. Timely identification of patients requiring NSICU admission may improve ED overcrowding and resource allocation.

You cannot alter accepted Supplementary Information files except for critical changes to scientific content. If you do resupply any files, please also provide a brief (but complete) list of changes. If these are not considered scientific changes, any altered Supplementary files will not be used, only the originally accepted version will be published.No Supplementary Information files need to be altered at the current time.

Driven by increases in electronic health data availability^13^ and computing power, machine learning is increasingly used to automate processes in healthcare^14–20^. While many such approaches make use of structured, or “tabular”, data, free text constitutes a large proportion of electronic health record (EHR) data^21^, and may capture greater clinical expressivity than tabular data^22–24^. Despite its richness, free text is vulnerable to the ambiguities of human language and is thus challenging to analyze. Natural language processing (NLP) can assist with such challenges by providing structure to free text^25^. While previous tabular data models^26–32^ have addressed the problems of reducing ED crowding, early disposition prediction, and unplanned ICU readmission^33^, other models have been developed using either a combination of free-text and tabular data^34^ or exclusively free-text data sources^35–37^. To our knowledge, NLP methods have not been employed to predict NSICU admission. We sought to develop a predictive model of NSICU admission at 30 min after arrival to the ED, using a combination of tabular and free text data.

## Methods

### Design, patient population and measurements

All research was performed in accordance with relevant guidelines and regulations of the Institutional Review Board of the Icahn School of Medicine at Mount Sinai, which approved this retrospective study under protocol number 19-02333, and waived the requirement of informed consent. We retrieved patient data from the Mount Sinai Hospital, an urban academic tertiary-care medical center located in New York City. Using our institutional data warehouse, we identified patients that presented to our ED between January 2014 and December 2018. From this cohort, we extracted tabular clinical and demographic data, which included age, sex, home address ZIP code, ethnicity, means of arrival, chief complaint, triage vital signs, patient escort, Emergency Severity Index^38^, medical history, number of previous hospitalization and ED visits, time since previous ED presentation, and admission/discharge/transfer records, which we used to identify patient bed movements. All variables were available in the EHR within 30 min of the patient’s arrival in the ED.

From the same data source, we also extracted all nurse and physician notes recorded up to 30 min from ED arrival. Upon a patient’s arrival in the Mount Sinai ED, the first clinical documentation recorded in a patient’s chart is a triage note consisting of structured chief complaint field and an abbreviated patient history written by a triage nurse. Therefore, the triage note time was used to designate ED arrival time. We derived continuous variables from tabular and free text data, including time from ED arrival to triage vital signs. Admission/discharge/transfer records were used to identify NSICU admission. We excluded all patients that were 17 years of age or younger at the time of ED presentation, or died within 30 min of arrival to the ED. We also excluded erroneously created or duplicate patient records, as well as visits that lacked an initial triage note.

### Model development

Using both tabular and free text data, we built a model to predict NSICU admission at 30 min from ED arrival time. While they constituted tabular data, admission/discharge/transfer records were used to identify the outcome of interest and therefore were not included as model predictors. Tabular data used in model training is represented in Supplemental Table 1. A bag-of-words (BOW) approach was used to represent the free text data. In a BOW model, a free text paragraph is represented as an unordered collection (or “bag”) of words. Words in the “bag” are organized into tabular representation according to frequency and number. A statistical classifier is then trained to classify each paragraph based on word frequency and number.

We first cleaned the free text data by lower-casing all words and removing punctuation and rare words. We then applied the bag-of-words (BOW) model to the clean text corpus. Both tabular data and BOW vectors were combined and incorporated into a gradient boosting model (XGBoost)^39^ to predict NSICU admission. All vectors were represented using sparse vector representations and the structured tabular vectors were directly concatenated to the unstructured vectors. Although the dimensionality of the structured vectors was much smaller than the unstructured vectors, due to the sparseness of the unstructured vectors, the latter occupied a relatively small amount of memory. Missing values were handled by the XGBoost model. Cohort imbalance was handled by the XGBoost weight scaling feature.

Model training was performed using data from January 1st, 2014 to December 31st, 2017. Model testing was performed using a hold-out dataset, which included data from January 1st, 2018 to December 31st, 2018. This was done to minimize model over-fitting and provide a pseudo-prospective estimate of model performance. We also separately determined the classifying ability of models exclusively trained using tabular data and free text, respectively. To ensure that our study exclusion criteria did not introduce selection bias, we performed a sensitivity analysis in which we derived all 3 models using the same methodology as the main analysis, but with a cohort population that included all patients who died within 30 min of ED arrival. To investigate whether using chronological testing-training data split introduced potential bias, we performed a second sensitivity analysis where all patient data was randomly split into 90% training and 10% testing sets. The same hyper-parameters were used in this sensitivity analysis as those used in the main analysis.

### Statistical analysis

To analyze word importance, we determined the mutual information (MI) between each word in the free text corpus and admission to the NSICU. MI measures the statistical dependence between two random variables^40–42^, such as an outcome (A) and a given word (w), according to the formula

$$\sum \sum P\left( {A,{ }w} \right){*}log{ }\frac{{P\left( {A,w} \right)}}{P\left( A \right)P\left( w \right)}$$

where P(A,w) represents the joint probability of (A) and (w), P(A) represents the probability of (A), and P(w) represents the probability of (w). To contrast the words associated with admission to the NSICU, we also determined the MI between each word and admission to non-NSICU services or discharge from the ED. For each word, we also determined the odds ratios (OR) associated with NSICU admission a swell as admission to non-NSICU services or ED discharge. We used the chi-squared test to evaluate the statistical significance of the associations between specific words and NSICU admission.

For all models, we constructed receiver operating characteristic (ROC) curves and evaluated the area under the ROC curve (AUC). We determined sensitivity, false-positive rate (FPR), positive predictive value (PPV), negative predictive value (NPV), F1 score, and Matthews correlation coefficient (MCC) for fixed model specificities of 0.90, 0.95, and 0.99. We characterized the relationship between all tabular variables and admission to the NSICU using univariate logistic regression, and determined AUCs corresponding to each variable. We reported the variables with the 10 highest AUC values. We determined the words with the 20 highest MIs with admission to the NSICU, and separately with admission to either non-NSICU services or discharge from the ED. Bootstrapping validations with 1,000 bootstrap re-samples were used to calculate 95% confidence intervals (CI) for all metrics. We used Youden's index to identify optimal sensitivity–specificity cutoff points for each ROC curve. Due to multiple comparisons, a Bonferroni correction was applied for controlling Type I error and Bonferroni-corrected alpha was set to 0.001.

## Results

**Figure 2:**
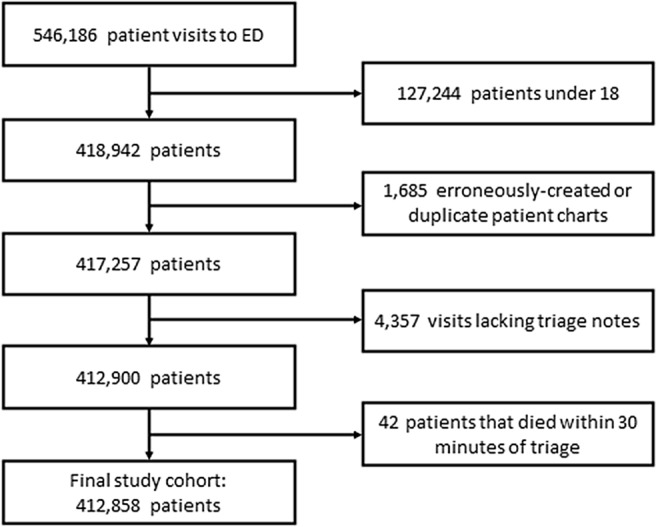
Study inclusion flowchart. *ED* emergency department, *NSICU* neuroscience intensive care unit.

**Figure 3:**
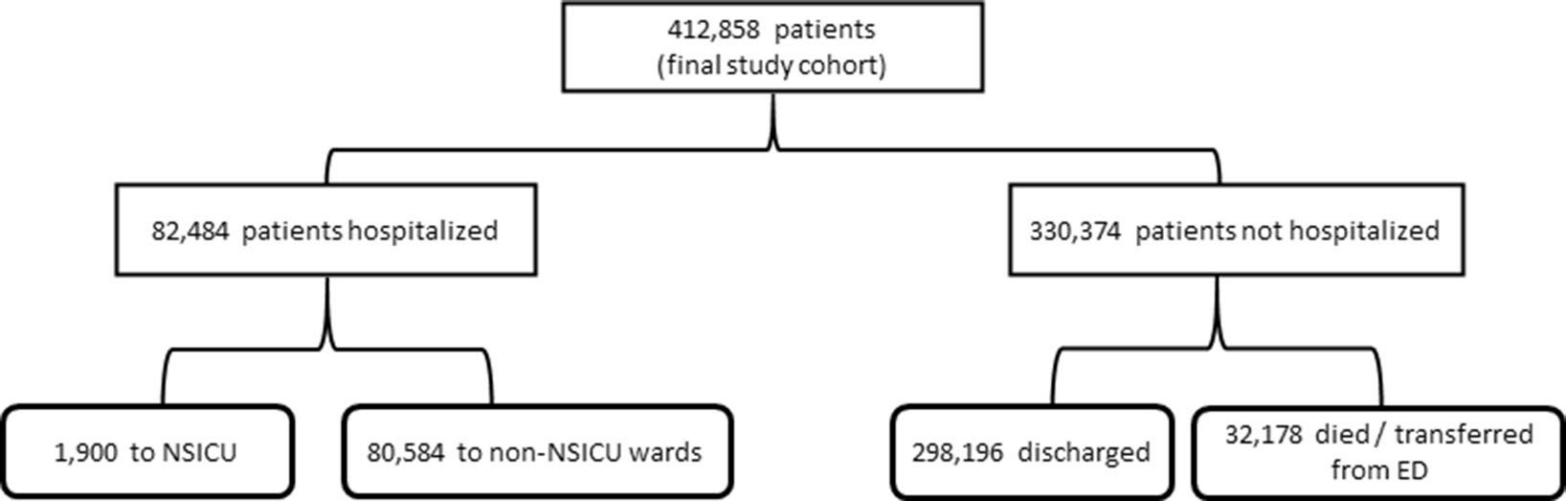
Cohort disposition. *NSICU* neuroscience intensive care unit.

Over the 5-year study period, we identified 412,858 patients after applying exclusions (Fig. 2), of whom 1900 (0.5%) were admitted to the NSICU (Fig. 3). The median number of ED visits was 231 (IQR 200–256), and the median time difference between ED arrival to the time of decision for NSICU admission was 169 min (IQR 80–324). Patients that were admitted to the NSICU were significantly more likely to be older, female, present with an “Immediate” (Level 1) or “Emergent” (Level 2) ESI, be transported to the ED by ambulance or emergency medical services, and have fewer preceding ED presentations than patients who were admitted to non-NSICU services or discharged from the ED. Patients admitted to the NSICU were also more likely to have higher triage systolic blood pressures, respiratory rates, and heart rates, and were also more likely to have a history of stroke, hypertension, cardiovascular disease, and neoplastic disease than patients who were admitted to non-NSICU services or discharged from the ED (Table 1).

**Table 1:** Clinical and demographic characteristics of patient cohort, stratified by admission to the neurosciences intensive care unit (NSICU).

Characteristic	Not Admitted to NSICU (N = 410,958)	Admitted to NSICU (N = 1900)	p value
**Demographics**			
Age, median (IQR), y	48.0 (30.0–63.0)	64.0 (54.0–75.0)	< 0.001
Female sex	239,662 (58.3)	951 (50.1)	< 0.001
Non-Hispanic ethnicity	229,257 (55.8)	1188 (62.5)	0.015
**Means of arrival to ED**			
Personal means	166,743 (40.6)	317 (16.7)	< 0.001
911 EMS	78,892 (19.2)	613 (32.3)	< 0.001
Private/non-911 EMS	20,687 (5.0)	475 (25.0)	< 0.001
**Patient escort**			
Self only	216,803 (52.8)	419 (22.1)	< 0.001
Family member	118,088 (28.7)	816 (42.9)	< 0.001
Unknown	76,067 (18.5)	665 (35.0)	< 0.001
**Emergency Severity Index**			
Immediate (1)	1615 (0.4)	170 (8.9)	< 0.001
Emergent (2)	86,128 (21.0)	1079 (56.8)	< 0.001
Urgent (3)	224,582 (54.6)	608 (32.0)	< 0.001
Less Urgent (4)	87,558 (21.3)	27 (1.4)	< 0.001
Non-Urgent (5)	9057 (2.2)	0 (0.0)	< 0.001
**Clinical characteristics**			
SBP, median (IQR), mmHg	132.0 (119.0–149.0)	140.0 (121.0–164.0)	< 0.001
DBP, median (IQR), mmHg	73.0 (65.0–82.0)	76.0 (65.0–89.0)	< 0.001
Heart rate per minute, median (IQR)	84.0 (74.0–96.0)	85.0 (72.0–100.0)	< 0.001
Temperature, median (IQR), °F	97.5 (96.8–98.2)	97.5 (96.8–98.3)	0.261
Respiratory rate per minute, median (IQR)	18.0 (18.0–20.0)	18.0 (18.0–20.0)	< 0.001
**Medical comorbidities**			
Stroke	13,158 (3.2)	337 (17.7)	< 0.001
Hypertension	114,364 (27.8)	583 (30.7)	0.006
Diabetes mellitus	101,645 (24.7)	473 (24.9)	0.892
Cardiovascular disease	23,055 (5.6)	162 (8.5)	< 0.001
Neoplastic disease	77,839 (18.9)	485 (25.5)	< 0.001
**Visit history**			
Prior ED visits, median (IQR)	1.0 (0.0–4.0)	0.0 (0.0–1.0)	< 0.001
Prior hospitalizations, median (IQR)	0.0 (0.0–0.0)	0.0 (0.0–1.0)	0.155

All figures reported as numbers (%) unless otherwise specified. Patients not admitted to NSICU include patients admitted to non-NSICU services, discharged from ED, or died/transferred in/from ED.

*NSICU* neurosciences intensive care unit, *IQR* interquartile range, *SBP* systolic blood pressure, *DBP* diastolic blood pressure, *ED* emergency department, *EMS* emergency medical services.

Free text words that had the highest MI with NSICU admission were related to stroke, including treatment (e.g. “tpa”), first-line neuroimaging (e.g. “ct”), symptoms (e.g. “droop”), alert notification (e.g. “activated”), and transfer from other hospitals (e.g. “transferred”) (Table 2). Words associated with the highest MI values with admission to a non-NSICU service or hospital discharge were related to pain, subacute complaints (e.g., “days”), or complaints referable to non-neurological organ systems (e.g., “sob”, “swelling”), as well as denial of symptoms (e.g., “denies”) (Supplemental Table 2). In the univariate analysis, the tabular variables with the highest AUC were chief complaint (AUC 0.85), followed by acuity (AUC 0.77), age (AUC 0.74), and means of arrival (AUC 0.73) (Table 3).

**Table 2:** Words with 10 highest mutual information values with NSICU admission and associated odds ratios.

Word	MI (× 10^y^)	OR	p value
Stroke	5.6	27.6	< 0.001
ct	4.8	12.6	< 0.001
Transferred	3.4	29.7	< 0.001
Team	3.3	20.0	< 0.001
Neuro	3.3	7.7	< 0.001
cva	3.0	10.1	< 0.001
tpa	2.9	116.2	< 0.001
From	2.9	3.7	< 0.001
Droop	2.9	24.8	< 0.001
Activated	2.8	41.2	< 0.001

All words are from clinician and nursing notes.

*NSICU* neurosciences intensive care unit, *MI* mutual information, *OR* odds ratio, *ct* computed tomography, *cva* cerebrovascular accident, *tpa* tissue plasminogen activator.

**Table 3:** Tabular variables with the 10 highest AUC contributions for admission to the NSICU.

Variable	AUC
Chief complaint	0.85
ESI	0.77
Age	0.74
Means of arrival	0.73
Address (ZIP code)	0.67
Patient escort	0.65
Number of previous ED visits	0.61
Time difference: first note to first respiratory rate measurement	0.61
Time difference: first note to first temperature measurement	0.61
Days to previous ED visit	0.61

*NSICU* neuroscience intensive care unit, *AUC* area under receiver-operating-characteristic curve, *ESI* Emergency Severity Index, *ZIP* zone improvement plan, *ED* emergency department.

**Figure 4:**
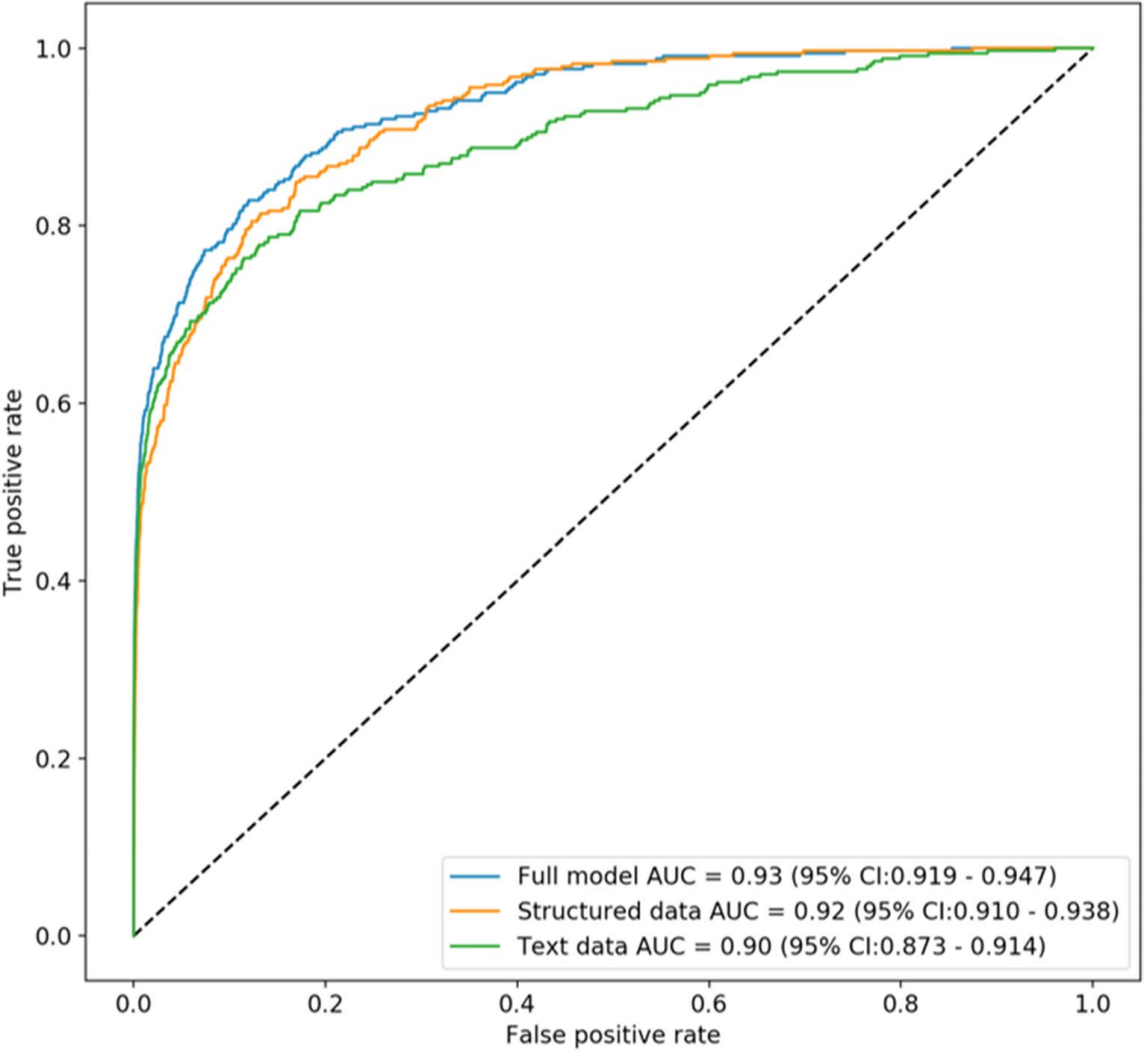
Receiver-operating curve for all 3 models. Combined model performance is illustrated in blue, tabular data-only model performance in orange, and text-only model in green. *AUC* area under receiver-operating curve.

Using tabular data alone, the model demonstrated an AUC of 0.92 (95% CI 0.91–0.94) (Supplemental Table 3), whereas the model trained solely with text generated an AUC of 0.90 (95% CI 0.88–0.92) (Supplemental Table 4). The final, combined model demonstrated an AUC of 0.93 (95% CI 0.92–0.95) (Fig. 4). At a false positive rate of 1:100 (99% specificity), the combined model was 58% sensitive for identifying NSICU admission, whereas at a false negative rate of 1:5 (80% sensitivity), the model was 88% specific for identifying NSICU admission (Table 4). In the first sensitivity analysis including patients that died within 30 min of ED arrival, the text data model showed an AUC of 0.89 (95% CI 0.87–0.91), the tabular data only model had an AUC of 0.92 (95% CI 0.91–0.94), and the combined model had an AUC of 0.94 (95% CI 0.92–0.95). In the second sensitivity analysis using a random data split, the text data model had an AUC 0.90 (95% CI 0.87–0.92), the tabular-only model showed an AUC 0.90 (95% CI 0.87–0.93), and the combined model showed 0.93 (95% CI 0.91–0.95).

**Table 4:** Combined model performance at fixed specificity cut points.

Specificity	FPR	Sensitivity	PPV	NPV	F1	MCC
0.88^a^	1:8	0.80 (0.76–0.85)	0.03 (0.03–0.04)	1.00 (1.00–1.00)	0.07 (0.06–0.07)	0.15 (0.14–0.16)
0.90	1:10	0.80 (0.76–0.85)	0.03 (0.03–0.04)	1.00 (1.00–1.00)	0.07 (0.06–0.07)	0.15 (0.14–0.16)
0.95	1:20	0.71 (0.66–0.76)	0.07 (0.06–0.07)	1.00 (1.00–1.00)	0.12 (0.11–0.13)	0.21 (0.19–0.22)
0.99	1:100	0.58 (0.53–0.63)	0.21 (0.19–0.24)	1.00 (1.00–1.00)	0.31 (0.28–0.34)	0.35 (0.31–0.38)

All metrics are reported as metric (95% CI) except for false-positive rates, which are reported as ratios.

*FPR* false positive rate, *PPV* positive predictive value, *NPV* negative predictive value, *MCC* Matthew’s correlation coefficient.

^a^Youden’s index.

## Discussion

We developed a machine learning model based on free text and tabular data to predict NSICU admission with good discriminatory performance. The model performance did not significantly change after we used a random training–testing data split and included patients that had been excluded from the study cohort, both of which suggest robust internal validity. Such a model could be used by neurocritical care experts and clinical stakeholders, such as ED clinicians and nursing managers, to identify patients that might need a NSICU bed early in the ED triage process. More importantly, this could also permit providers to administer timely neurocritical care therapies, accelerate patient movement into the NSICU, and potentially reduce ED boarding time.

We found that the NSICU admission rate from our ED was low, which likely explains the low positive predictive value of our model. As such, the task of distinguishing patients that require NSICU admission from other patients in the ED constitutes a “needle in a haystack” problem. Nonetheless, by using EHR data and machine learning approaches, our model can predict the need for NSICU admission within 30 min of arrival in triage with a reasonable tradeoff between false-positive and false-negative rates. Furthermore, considering that the median time from ED arrival to the decision for NSICU admission was 169 min over the study period, our model’s ability to generate a disposition prediction within 30 min of ED arrival could potentially translate to operational and clinical benefits.

The free text data corpus for our model consisted of all physician and nursing notes documented up to 30 min from ED arrival. Our model’s short predictive time window and extent of included text data builds on prior NLP-based approaches aimed at predicting patient disposition from the ED. For example, Lucini et al.^36^ incorporated provider notes available several hours after patient arrival, and Zhang et al.^37^ used text taken from patients’ chief complaints. Sterling et al. demonstrated that triage notes were useful predictors of ED disposition when used as the sole data source^35^. Our findings suggest that prediction of NSICU admission is feasible within 30 min of ED arrival, and that combining tabular and free text data can slightly improve overall model performance over tabular data alone. However, incorporating text data into a predictive model for NSICU admission provided limited incremental benefit in performance over a tabular-only model, suggesting that tabular data may represent overlapping predictive features with those denoted by highly predictive keywords identified using NLP. From an implementation perspective, this modest benefit in model performance should be weighed against any computing requirements or technical costs of using NLP to incorporate textual data into a combined predictive model.

Our analysis of ED notes suggests that words most highly associated with NSICU admission related directly to acute cerebrovascular events, such as stroke or symptoms thereof, and hospital transfers. By contrast, words associated with admission to non-NSICU services or ED discharge suggested non-acute illnesses and non-neurological complaints. While many neurological and non-neurological emergencies are managed in NSICUs^43^, these findings are expected. Acute cerebrovascular diseases are commonly encountered in the ED and frequently require NSICU care, especially in cases of large-vessel arterial occlusion that necessitate mechanical thrombectomy. Our institutional ED is a frequent referral destination in a New York City-wide network for emergent endovascular thrombectomy in acute ischemic stroke, thereby also explaining some of the findings from our text analysis^44, 45^. However, because clinical factors such as neurological examination findings may be the main driver for identifying patients who require NSICU admission, the keywords we have identified may provide limited predictive benefit over existing decision making approaches.

One potential implementation of such a model could be a clinical decision support tool that identifies patients requiring NSICU admission, and immediately delivers a notification to specialized neurocritical care teams. In such an implementation, selection of an optimal alert threshold necessitates careful evaluation of model performance, and likely depends on multiple factors, including healthcare institution needs, clinical stakeholder preferences, and NSICU resource availability. In many institutions, such as ours, the costliness of NSICU resource mobilization may justify a model threshold that seeks to minimize false positive notifications.

At a false positive rate of 1:100, the false-negative rate of our model was 42%. Consistent with stroke care system guidelines^46^, and like many other stroke-capable hospitals, Mount Sinai possesses a stroke notification paging system that alerts clinicians of patients with suspected stroke in the ED and hospital floors. This system is frequently used for patients with acute neurological signs or symptoms irrespective of cerebrovascular etiology. This system could therefore be used in combination with our predictive model as to potentially minimize type II error in identifying patients that require NSICU admission.

### Limitations

Our study was limited by a number of notable factors. First, this was a single-center, retrospective study. Although our medical center treats a diverse urban population, our analysis incorporated multiple, potentially non-generalizable factors, such as a specific staffing configurations, resource availability, triage procedures, and practice styles. For instance, in many institutions such as ours, admission to a dedicated stroke unit is standard practice following administration of tissue plasminogen activator for acute ischemic stroke, whereas in other institutions lacking a stroke unit, the same clinical scenario requires admission to the NSICU for post-thrombolysis monitoring. Therefore, it is important to consider that the classifying ability of our model for NSICU admission may decrease when tested or deployed in settings comprising different setting-specific factors. Second, biases exist in the documentation technique and content of triage notes used in the model. Third, we did not compare the performance of our model to that of trained clinicians, and we did not operationalize our model, which prevented us from assessing the feasibility of implementing our model and supporting care decisions. Fourth, we handled the free text data in our model by using BOW modeling, which does not capture word order. Alternative NLP methods, such as distributional embeddings or neural networks, may provide better results than those in our study. However, despite its limitations, BOW has the advantages of being simple to use, infer, implement, and integrate with tabular data.

## Conclusions

We developed a model to predict admission to the NSICU within 30 min of ED arrival using free text and tabular data with good discriminatory performance. Our results suggest that NLP can be used to combine text with tabular data, although such a combination only afforded a marginal improvement in overall discriminatory performance over tabular-only models. Despite the discriminatory performance demonstrated in this study, specificity and sensitivity thresholds should be guided by institutional priorities and preferences, and our findings should be validated in other patient cohorts. Future plans include implementation of this model as a clinical decision support tool, and prospectively comparing the triage our model’s performance against that of unassisted neurocritical care experts.

## Supplementary Information

Supplementary Information
