# Supplementary material for: Predicting adult neuroscience intensive care unit admission from emergency department triage using a retrospective, tabular-free text machine learning approach
Source: Sci Rep. 2021 Jan 14;11:1381. doi: 10.1038/s41598-021-80985-3 (PMC7809037; doi:10.1038/s41598-021-80985-3)
Supplement: Supplementary file 1 — Supplementary Information [file 41598_2021_80985_MOESM1_ESM.pdf]

Supplementary Data

Predicting Neuroscience Intensive Care Unit Admission in Adult Patients at Emergency Department Triage Using a Retrospective, Tabular-Free Text Machine Learning Approach

Eyal Klang MD†, Benjamin R. Kummer MD†, Neha S. Dangayach MD MSCR, Amy Zhong MA, M. Arash Kia MD MSc, Prem Timsina ScD, Ian Cossentino BS, Anthony B. Costa PhD, Matthew A. Levin MD, Eric K. Oermann MD

† Co-first authors

Supplemental Table 1. Tabular Data Used in Model Training.

Data point	Variable type
Age	Continuous
ZIP code	Categorical
Sex	Categorical
Ethnicity	Categorical
ED presentation date (day)	Categorical
ED presentation date (month)	Categorical
ED presentation date (year)	Categorical
Means of arrival to ED	Categorical
Patient escort	Categorical
Acuity level by ESI	Categorical
Chief complaint	Categorical
Systolic blood pressure	Continuous
Diastolic blood pressure	Continuous
Heart rate	Continuous
Temperature	Continuous
Respirations	Continuous
Pulse oximetry	Continuous
Time from triage to first vital sign	Continuous
Number of past ED visits	Continuous
Number of past hospital admissions	Continuous
Number of past NSICU admissions	Continuous
Days since last ED visit	Continuous
Days since last admission	Continuous
Days since last NSICU admission	Continuous
Medical comorbidities	Categorical

Abbreviations: ZIP, Zone Improvement Plan; ED, emergency department; NSICU, neurosciences intensive care unit.

Supplemental Table 2. Words with 10 Highest Mutual Information Values with Admission to Non-NSICU Services or Hospital Discharge

Word	MI (x 10^3)	OR	p value
co	1.4	2.3	<0.001
pain	1.3	2.2	<0.001
denies	0.8	1.8	<0.001
days	0.5	1.5	<0.001
states	0.4	1.5	<0.001
sob	0.3	1.3	<0.001
swelling	0.2	1.3	<0.001
injury	0.2	1.9	<0.001
location	0.2	2.2	<0.001
fever	0.2	1.2	0.01

All words are from clinician and nursing notes.
Abbreviations: NSICU, neurosciences intensive care unit; MI, mutual information; OR, odds ratio.

Supplemental Table 3. Tabular-only Model Performance at Fixed Specificity Cut-points

Specificity	FPR	Sensitivity	PPV	NPV	F1	MCC
0.88 [a]	1:8.3	0.80 (0.75 - 0.84)	0.03 (0.03 - 0.03)	1.00 (1.00 - 1.00)	0.06 (0.05 - 0.07)	0.14 (0.13 - 0.15)
0.90	1:10	0.76 (0.72 - 0.81)	0.03 (0.03 - 0.04)	1.00 (1.00 - 1.00)	0.07 (0.06 - 0.07)	0.15 (0.14 - 0.16)
0.95	1:20	0.66 (0.61 - 0.71)	0.06 (0.05 - 0.07)	1.00 (1.00 - 1.00)	0.11 (0.09 - 0.12)	0.18 (0.17 - 0.20)
0.99	1:100	0.49 (0.43 - 0.53)	0.19 (0.16 - 0.22)	1.00 (1.00 - 1.00)	0.27 (0.24 - 0.30)	0.30 (0.26 - 0.33)

All metrics are reported as metric (95% CI) except for false-positive rates, which are reported as ratios.
Abbreviations: FPR, false positive rate; PPV, positive predictive value; NPV, negative predictive value; MCC, Matthew’s correlation coefficient.
[a] Youden’s index.

Supplemental Table 4. Text-only Model Performance at Fixed Specificity Cut-points

Specificity	FPR	Sensitivity	PPV	NPV	F1	MCC
0.89 [a]	1:9	0.76 (0.72 - 0.81)	0.03 (0.03 - 0.03)	1.00 (1.00 - 1.00)	0.06 (0.05 - 0.06)	0.13 (0.12 - 0.14)
0.90	1:10	0.72 (0.67 - 0.77)	0.04 (0.03 - 0.04)	1.00 (1.00 - 1.00)	0.07 (0.06 - 0.08)	0.15 (0.13 - 0.16)
0.95	1:20	0.67 (0.62 - 0.72)	0.06 (0.05 - 0.07)	1.00 (1.00 - 1.00)	0.11 (0.09 - 0.12)	0.19 (0.17 - 0.21)
0.99	1:100	0.53 (0.48 - 0.59)	0.20 (0.18 - 0.23)	1.00 (1.00 - 1.00)	0.29 (0.26 - 0.32)	0.32 (0.29 - 0.36)

All metrics are reported as metric (95% CI) except for false-positive rates, which are reported as ratios.
Abbreviations: FPR, false positive rate; PPV, positive predictive value; NPV, negative predictive value; MCC, Matthew's correlation coefficient.
[a] Youden's index.
